# Identification of CDK2-Related Immune Forecast Model and ceRNA in Lung Adenocarcinoma, a Pan-Cancer Analysis

**DOI:** 10.3389/fcell.2021.682002

**Published:** 2021-07-30

**Authors:** Ting-Ting Liu, Rui Li, Chen Huo, Jian-Ping Li, Jie Yao, Xiu-li Ji, Yi-Qing Qu

**Affiliations:** ^1^Department of Pulmonary and Critical Care Medicine, Qilu Hospital, Cheeloo College of Medicine, Shandong University, Jinan, China; ^2^Shandong Key Laboratory of Infectious Respiratory Diseases, Jinan, China; ^3^Department of Pulmonary Disease, Jinan Traditional Chinese Medicine Hospital, Jinan, China; ^4^Department of Respiratory and Critical Care Medicine, Qilu Hospital of Shandong University, Jinan, China

**Keywords:** tumor microenvironment, CDK2, pan-cancer analysis, nomogram model, prognostic model, ceRNA

## Abstract

**Background:**

Tumor microenvironment (TME) plays important roles in different cancers. Our study aimed to identify molecules with significant prognostic values and construct a relevant Nomogram, immune model, competing endogenous RNA (ceRNA) in lung adenocarcinoma (LUAD).

**Methods:**

“GEO2R,” “limma” R packages were used to identify all differentially expressed mRNAs from Gene Expression Omnibus (GEO) and The Cancer Genome Atlas (TCGA) databases. Genes with *P*-value <0.01, LogFC>2 or <-2 were included for further analyses. The function analysis of 250 overlapping mRNAs was shown by DAVID and Metascape software. By UALCAN, Oncomine and R packages, we explored the expression levels, survival analyses of CDK2 in 33 cancers. “Survival,” “survminer,” “rms” R packages were used to construct a Nomogram model of age, gender, stage, T, M, N. Univariate and multivariate Cox regression were used to establish prognosis-related immune forecast model in LUAD. CeRNA network was constructed by various online databases. The Genomics of Drug Sensitivity in Cancer (GDSC) database was used to explore correlations between CDK2 expression and IC50 of anti-tumor drugs.

**Results:**

A total of 250 differentially expressed genes (DEGs) were identified to participate in many cancer-related pathways, such as activation of immune response, cell adhesion, migration, P13K-AKT signaling pathway. The target molecule CDK2 had prognostic value for the survival of patients in LUAD (*P* = 5.8e-15). Through Oncomine, TIMER, UALCAN, PrognoScan databases, the expression level of CDK2 in LUAD was higher than normal tissues. Pan-cancer analysis revealed that the expression, stage and survival of CDK2 in 33 cancers, which were statistically significant. Through TISIDB database, we selected 13 immunodepressants, 21 immunostimulants associated with CDK2 and explored 48 genes related to these 34 immunomodulators in cBioProtal database (*P* < 0.05). Gene Set Enrichment Analysis (GSEA) and Metascape indicated that 49 mRNAs were involved in PUJANA ATM PCC NETWORK (ES = 0.557, *P* = 0, FDR = 0), SIGNAL TRANSDUCTION (ES = –0.459, *P* = 0, FDR = 0), immune system process, cell proliferation. Forest map and Nomogram model showed the prognosis of patients with LUAD (Log-Rank = 1.399e-08, Concordance Index = 0.7). Cox regression showed that four mRNAs (SIT1, SNAI3, ASB2, and CDK2) were used to construct the forecast model to predict the prognosis of patients (*P* < 0.05). LUAD patients were divided into two different risk groups (low and high) had a statistical significance (*P* = 6.223e-04). By “survival ROC” R package, the total risk score of this prognostic model was AUC = 0.729 (SIT1 = 0.484, SNAI3 = 0.485, ASB2 = 0.267, CDK2 = 0.579). CytoHubba selected ceRNA mechanism medicated by potential biomarkers, 6 lncRNAs-7miRNAs-CDK2. The expression of CDK2 was associated with IC50 of 89 antitumor drugs, and we showed the top 20 drugs with *P* < 0.05.

**Conclusion:**

In conclusion, our study identified CDK2 related immune forecast model, Nomogram model, forest map, ceRNA network, IC50 of anti-tumor drugs, to predict the prognosis and guide targeted therapy for LUAD patients.

## Introduction

Lung cancer is the third leading cause of death in the world (Siegel et al., 2020; Kara et al., 2021), which is classified into small cell lung cancer (SCLC), lung squamous cell carcinoma (LUSC) and lung adenocarcinoma (LUAD) (Wu Y. et al., 2021). Non-small cell lung cancer (NSCLC) accounted for 85% of lung cancer and the 5-year survival rate of the patients is less than 20% (Bade and Dela Cruz, 2020). LUAD is the most important type of NSCLC (Chen and Zhou, 2021; Hou and Yao, 2021; Zhou C. et al., 2021). In recent years, although the treatment of lung cancer is diversified, such as chemotherapy, immunotherapy, targeted therapy, the prognosis of patients with advanced lung cancer is still poor (Noreldeen et al., 2020; Wang et al., 2020). More than 60% of LUAD patients missed the best targeted treatment time due to the difficulty of diagnosis, which can reduce the survival rate (Kris et al., 2014; Chen et al., 2016). In recent years, the resistance of the majority of LUAD patients to multiple antitumor drugs has led to a decrease in the cure rate of LUAD. Therefore, it is very necessary to explore the early gene markers and treatment targets for the prognosis of patients (He et al., 2021).

The immune system is currently recognized as a determinant of cancer (Janssen et al., 2017). With the development of modern technology, new immunotherapy drugs have made remarkable achievements and improved the prognosis of patients (Yang et al., 2021). Combined with various clinical studies, immunotherapy may replace the traditional treatment (Zhai et al., 2021). Understanding TME and recognizing genes related to TME can provide new ideas for immunotherapy. In the TME, many cancer cells and immune cells mediate signaling pathways (Marwitz et al., 2021), involving in tumor progression and drug resistance (Santaniello et al., 2019; Ling et al., 2020). In view of the low treatment rate of LUAD patients with high pathogenicity, it is imperative to find new gene markers (Zhou C. S. et al., 2021).

The mechanism of immune infiltration plays an irreplaceable role in the progress of various cancers (Esenboga et al., 2021; Huang H. et al., 2021; Jomrich et al., 2021; Mungan et al., 2021). The novel systemic immune-inflammation index (SII) is a new kind of marker, including peripheral lymphocytes, neutrophils, and platelets (Ju et al., 2021). SII features play important roles in various cancers, such as Esophageal Squamous Cell Carcinoma (Geng et al., 2016), hepatocellular carcinoma (Hu et al., 2014), and prostate cancer (Lolli et al., 2016).

As for a non-coding RNA, lncRNA was limited to code protein (Chen and Zhang, 2021). In recent years, there has been increasing evidence that the expression of lncRNA involves in cancer progression, such as cancer metastasis (Li et al., 2016; Kim et al., 2018), drug resistance (Han et al., 2017), and apoptosis (Zhao et al., 2017). ceRNA network was composed of mRNAs, lncRNAs, and miRNAs (Chen Y. et al., 2021). lncRNA MALAT1 modulates cell migration, proliferation by sponging miRNA146a to regulate CXCR4 in acute myeloid leukemia (Sheng X. F. et al., 2021). ceRNA network represents a novel layer of gene regulation that controls both physiological and pathological processes (Zhang et al., 2021).

Our study aims to explore immune-related genes and construct forecast model for clinical guidance and prognosis analysis in LUAD using TCGA and GEO common databases. The relationship between target genes and immune cells is studied through various authoritative databases. We built the lncRNAs-miRNAs-CDK2 ceRNA network innovatively, found potential prognostic markers with LUAD. Our study may provide new molecular and therapeutic strategies for the treatment and prognosis of LUAD patients.

## Materials and Methods

### Gene Expression mRNA Seq, Clinical Data Collection

We downloaded the gene expression, clinical data, Pan-Cancer Atlas Hub from GEO database^1^ (Chen C. et al., 2021), UCSC Xena^2^ (Li F. et al., 2021), GDSC^3^.

### mRNA-Based Survival Prediction in LUAD

Four hundred and thirty three LUAD tissues and 19 normal tissues were selected from the GSE68465 (GPL96). By GEO2R (Liu et al., 2019), *P* < 0.01, Log FC > 2 or Log FC < -2 were defined as screening criteria. Next, Metascape^4^ (Liu et al., 2020), KEGG^5^ (Zheng et al., 2021), and DAVID^6^ (Li J. et al., 2021) databases were used to analyze biological process (BP), cellular component (CC), molecular function (MF) and related pathways of differentially expressed mRNAs (DEmRNAs).

### Validation of CDK2 in Different Databases

UALCAN^7^ (Liu H. et al., 2021) is a comprehensive, user-friendly, and interactive web resource for analyzing cancer OMICS data. Combing with clinical data, we explored the correlation between CDK2 expression and clinical indicators, such as age, grade, sex, smoking habits, stage, TP-53 mutation, weight. Heat maps were showed the positive and negative related genes with CDK2. We analyzed the overall survival (OS) and Relapse-Free Survival (RFS) about CDK2 for LUAD patients by PrognoScan^8^ (Liu X. et al., 2021).

### Further Study About CDK2 Expression in 33-Cancers: Pan-Cancer Analysis

Compared with normal lung tissues, CDK2 expression was highly expressed (*P* < 0.05) in LUAD by Oncomine^9^ (Dai et al., 2021) and TIMER^10^ (Wu R. et al., 2021) databases. As for 33-cancers, we studied the relationships between the expression of CDK2 and clinical stage, survival conditions through different “R” packages.

### Batch Related Genes, ROC Analysis, Functional Enrichment, GSEA Analysis in LUAD

To research the major molecule CDK2, we showed 50 positive and negative genes in LUAD by heat maps (Person test method). The 1, 3, 5, and 8 years’ ROC curves were constructed by ‘‘ROC’’ R package. The abscissa of the prediction model was False Positive (FP) and the ordinate was True Positive (TP). Next, we screened out the top 300 genes with the most significant positive correlation with CDK2 for enrichment analysis. Bar and bubble charts showed the classical functions of 300 genes by ‘‘cluster-profiler’’ R package. We downloaded the GSEA4.1.0^11^ (Cao et al., 2021a) to investigate Kyoto Encyclopedia of Genes and Genomes (KEGG) of CDK2-related 300 genes. The top of 50 terms were sorted by *P*-value in circle graph.

### Immune Infiltration of CDK2 in LUAD

CIBERSORT^12^ (Tan et al., 2021) is an analysis tool to provide an estimation of the abundances of member cell types in a mixed cell population using gene expression data. We selected immune cells associated with CDK2 (Pearson correlation coefficient *r* > 0.15). Four CDK2-related immune cells were shown in circle graph.

### Correlation Analysis of Immune Cells and CDK2 Expression

R package was used to analyze the relation of CDK2 and immune cell infiltration score (*P* ≤ 0.05). A total of 33-cancers, according to the median of CDK2 gene expression, the samples were divided into high expression groups and low expression groups. Then, we discussed the different expression of immune cells (*P* < 0.05). The above results were shown by scatter plots and box plots.

### Exploring CDK2-Related Immune Genes and GSEA Analysis

TISIDB^13^ (Huang X. Y. et al., 2021) is a web portal for tumor and immune system interaction, which integrates multiple heterogeneous data types. First, we found CDK2-related Immunomodulators with *P*-value < 0.05. Next, cBioPortal^14^ (Lu et al., 2021) was used to explore the genes related to Immunomodulators. DAVID and STRING^15^ (Zhuang et al., 2021) were used to analyze the Gene Ontology (GO), protein interaction of 49 genes. GSEA 4.1.0 further analyzed the NOM *p*-val and FDR *q*-val of these immune related genes.

### Combining Clinical Data and Constructing Nomogram Model

Through LUAD clinical data, “survival,” “survminer” R packages were used to construct the COX regression model of age, gender, and stage. Forest plot (Xiang et al., 2021) was shown related Hazard ratio (HR), Log-Rank, Concordance index of LUAD. “rms” R package was used to structure the Nomogram model (Liu et al., 1976) of age, gender, stage, T, N, M.

### Univariate, Multivariate Cox Regression and Time ROC Analysis

Using “Survival” R package, we explored the univariate Cox analysis of 49 genes (*P* < 0.05). Different *P*-value and HR of statistically significant genes were shown in forest map. According to the results of univariate regression analysis, the significant genes were further analyzed by multivariate analysis. According to the risk score of genes selected by multiple factors, the LUAD samples were divided into lower-risk and higher-risk groups. The risk score was as follows: Risk score = Expgene1 × Coefgene1+Expgene2 × Coefgene2+ Expgene3 × Coefgene3+ Expgene4 × Coefgene4. “survival,” “survminer,” “survival ROC” R packages were performed survival probability curve and ROC curve. Combining the patient’s high and low risk situation and the state of life, death, we used “pheatmap” R package to show the heat map.

### The CDK2 Expression Was Further Verified by Histochemistry

We use Human Protein Atlas (HPA) database to verify CDK2 expression in LUAD tissues and normal lung tissues.

### Identification of CDK2 Related ceRNA Network

We explored CDK2 related miRNAs from Targetscan^16^ (Barnett-Itzhaki et al., 2021), miRWalk^17^ (Sheng L. P. et al., 2021), mirDB^18^ (Ren et al., 2021), Starbase^19^ (Li et al., 2014). All miRNAs from four databases were analyzed by Venn map^20^. Kaplan–Meier Plotter software was used to explore the prognostic value of miRNAs with LUAD. The lncRNAs that regulate common miRNAs were screened by Starbase database (Li et al., 2014). The Cytoscape (Han et al., 2021) was used to build ceRNA network and Cytohubba selected the key node genes according to the degree in the network.

### The Expression of CDK2 Was Verified by Polymerase Chain Reaction (PCR)

We did the basic experimental verification of PCR about CDK2 in LUAD cell lines (A549, H1299, H1975) and normal bronchial epithelial cell line (BEAS-2B). GraphPad Prism7^21^ software was used to count the differences between cancer cell lines and normal cell line.

### Correlation Between CDK2 and IC50 of Anti-tumor Drugs

We downloaded the response data of 192 anti-tumor drugs in more than 1000 cancer cell lines. “Ggplot2” R package was used to explore the correlation between the CDK2 expression and IC50 of 192 anti-tumor drugs by box diagrams and point diagrams.

## Results

### Differentially Expressed mRNAs for GSE68465

**FIGURE 1:**
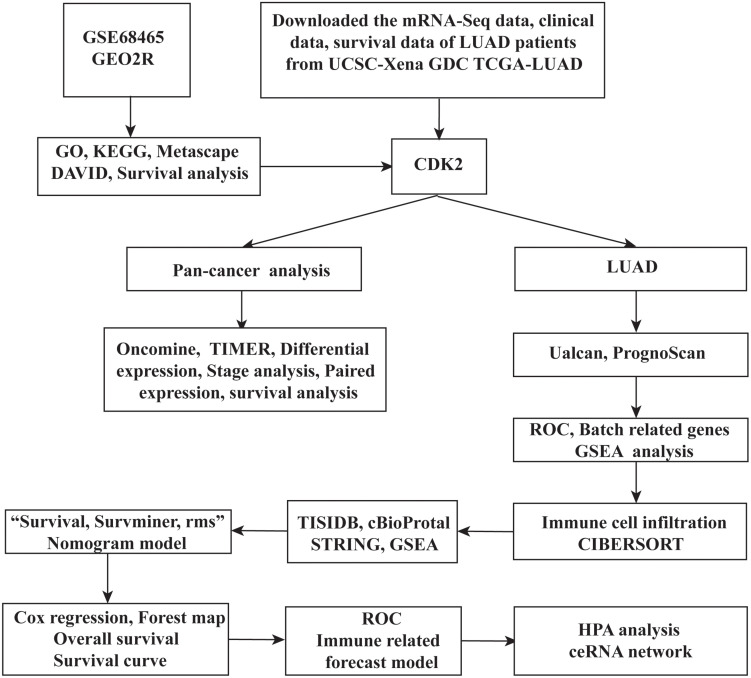
The overall research ideas of this paper were shown as follows.

**FIGURE 2:**
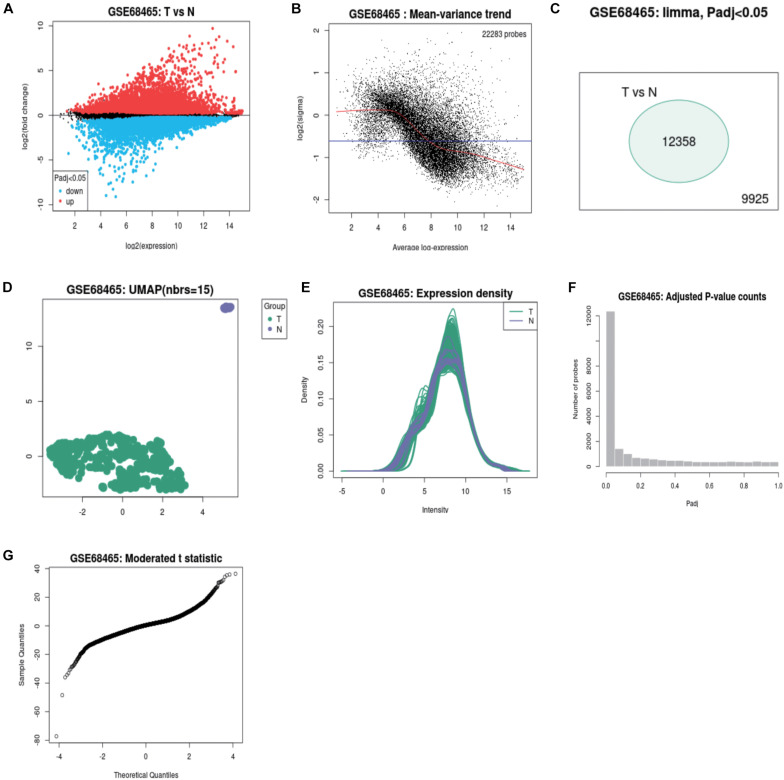
The volcano map, scatter plot, peak plot of GSE68465 samples **(A–G)**.

A total of 250 genes were chosen by GEO2R according to the *P-*value and LogFC. 161 mRNAs were highly expressed and 78 mRNAs were expressed at lower levels in 19 adjacent non-LUAD tissues and 433 LUAD tissues. The study design of this project was shown in Figure 1. The volcano map, scatter plots, peak plot of GSE68465 samples were displayed in Figure 2. A total of 15 highly expressed genes and 15 low expressing genes were shown in Table 1.

**TABLE 1:** The top of 15 higher expressed and 15 lower expressed genes.

Gene.symbol	logFC	adj.*P*.Val	*P*.Value
HLA-DRA	9.71	4.20E-134	5.65E-138
COL3A1	7.43	2.84E-132	6.37E-136
VWF	5.56	5.15E-130	1.39E-133
IGHG1	7.66	1.34E-125	4.79E-129
IGHA2	8.28	4.27E-116	1.92E-119
RGS1	8.84	1.47E-91	1.91E-94
IGK	4.9	4.78E-111	2.79E-114
COL3A1	6.72	2.56E-110	1.61E-113
HLA-DRB1	6.25	5.15E-109	3.46E-112
IGKC	4.86	1.02E-108	7.33E-112
CDH5	5.3	3.64E-108	2.78E-111
COL3A1	6.44	1.42E-98	1.40E-101
HLA-DRB5	5.9	1.88E-94	2.11E-97
C1QA	6.8	2.89E-94	3.37E-97
HLA-DPA1	5.79	6.27E-93	7.88E-96
HBZ	–9.92	1.40E-262	6.29E-267
DCT	–4.69	1.02E-179	9.14E-184
HBG2	–6.77	9.77E-133	1.75E-136
ALAS2	–4.84	8.02E-126	2.52E-129
HBG2	–9.11	1.26E-120	5.10E-124
HBG1	–6.15	3.06E-111	1.65E-114
HBE1	–7.47	4.18E-108	3.38E-111
AFP	–6.52	1.69E-101	1.44E-104
DCT	–4.83	1.76E-101	1.58E-104
AHSG	–8.98	5.18E-99	4.88E-102
SOX10	–4.59	6.18E-98	6.38E-101
DCT	–7.69	7.40E-95	7.97E-98
PMEL	–5.2	1.66E-93	2.01E-96
AHSG	–7.56	2.57E-89	3.58E-92
NANOG	–6.52	4.60E-86	6.81E-89

### GO, KEGG Analysis of 250 DEmRNAs

**FIGURE 3:**
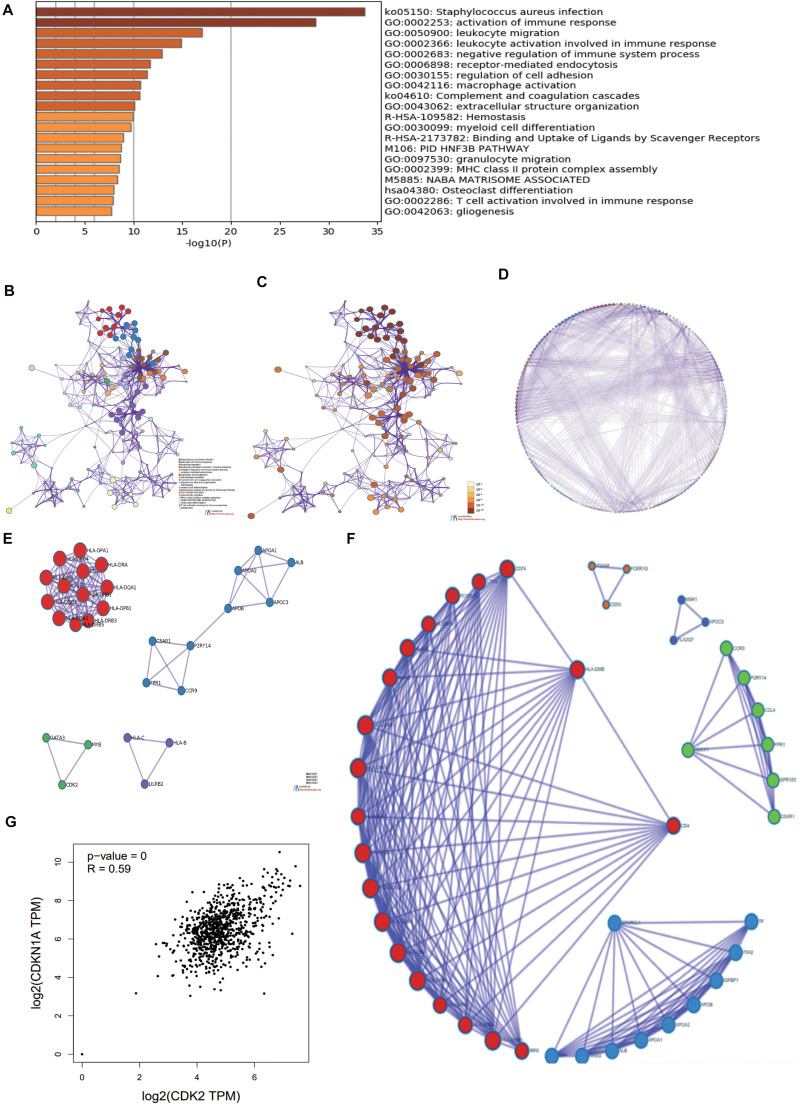
The GO, KEGG analysis of 250 DEmRNAs. **(A)** Through Metascape software, we found most enriched items. **(B)** The related paths were shown in the network diagram. **(C,D)** the *P*-value of different pathways. **(E)** According to the relationship of DE mRNAs, several protein analyses were performed. **(F)** CDK2, MYB, GATA3 were connected each other in some tumor pathway from the circle graph. **(G)** There was a positive correlation between CDK2 and CDKN1A using GEPIA.

Through Metascape software, we identified a number of pathways significantly enriched, including activation of immune response, regulation of cell adhesion, T cell activation involved in immune response, regulation of cell adhesion, PID-HNF3B PATHWAY (Figure 3A). The interactions between different pathways were shown in Figure 3B. Figures 3C,D drew the *P*-value of different pathways. According to the relationship of DE mRNAs, several protein analyses were performed in the Figure 3E. CDK2, MYB, GATA3 related to each other in some tumor pathway and the results were listed in circle graph (Figure 3F). DAVID was used to analyze the KEGG results of genes and we found 9 mRNAs participated in the classic P13-AKT signaling pathway, such as MYB, COL3A1, COL4A1, CSF1R, CDK2, ITGB7, LAMA2, TLR2, and VWF. Combining with the Overall Survival (OS) of LUAD patients, we chose CDK2 as targeted molecule (*P* < 0.05). KEGG software further was used to identify the upstream molecules of CDK2 in P13-AKT signaling pathway. Finally, the CDKN1A might regulate downstream molecule CDK2 to influence cell cycle progression in LUAD. Through the scatter plot, there was a positive correlation between CDK2 and CDKN1A using GEPIA^22^ (*P* = 0, *R* = 0.59) (Figure 3G).

### Further Study of CDK2 and Its Prognostic Values in LUAD

**FIGURE 4:**
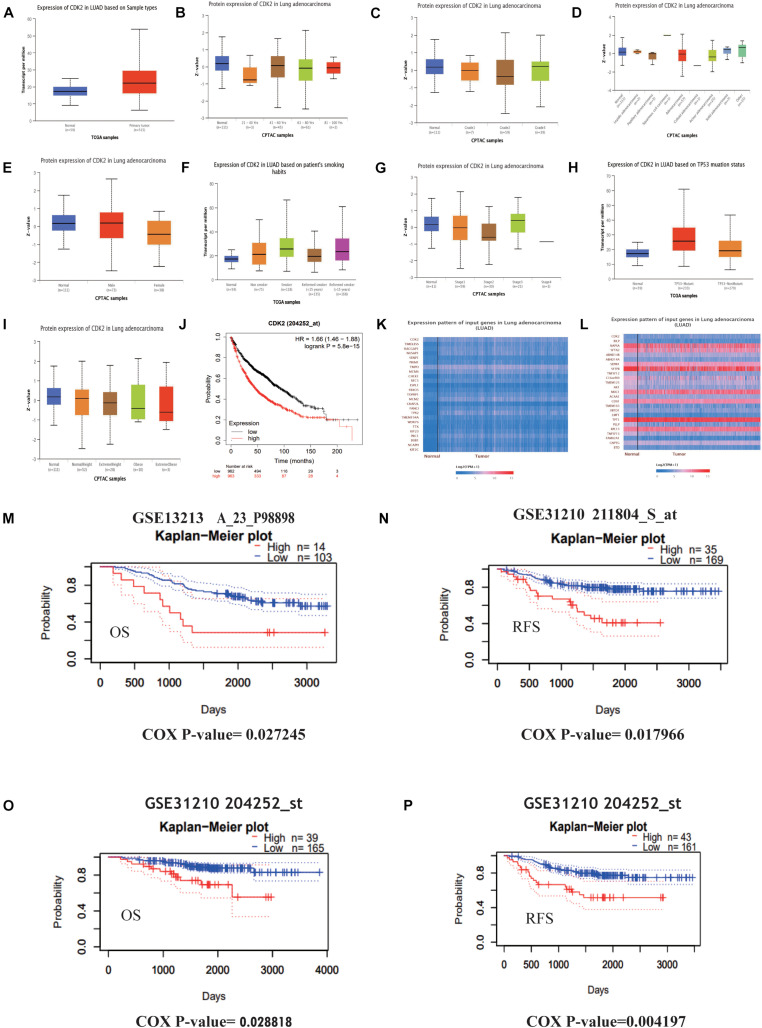
Further study of CDK2 and prognosis analysis in LUAD. **(A)** The expression of CDK2 was highly in 515 LUAD tissues than 59 normal tissues. **(B)** Different ages might have differential expression levels of CDK2. **(C)** The expression of CDK2 was associated with Grade. **(D)** The types of adenocarcinoma. **(E)** Gender. **(F)** Smoking habits. **(G)** Stage. **(H)** TP-53 mutation state. **(I)** Weight. **(J)** The expression of CDK2 was related to the survival and prognosis of patients with LUAD. **(K,L)** CDK2-related genes and heat maps. **(M–P)** The expression of CDK2 influenced the OS and RFS of LUAD patients.

Through the Ualcan database, we studied the expression of CDK2 in LUAD tissues and normal tissues. CDK2 expression was high in 515 LUAD tissues in comparison with 59 normal tissues (*P* = 1.624E-12) (Figure 4A). Different ages had differential expression levels of CDK2 (Normal-vs.-Age 61–80, *P* = 1.044E-2) (Figure 4B). The expression of CDK2 was associated with various clinical features, such as Grade (Normal-vs.-Grade2, *P* = 5.088808E-03) (Figure 4C), the types of adenocarcinoma (Normal-vs.- Adenocarcinoma, *P* = 2.195E-02) (Figure 4D), gender (Male-vs.-Female, *P* = 1.338E-03) (Figure 4E), smoking habits (Normal-vs.-Smoker, *P* = 1.319E-11) (Figure 4F), stage (Normal-vs.-stage1, *P* = 3.458E-02) (Figure 4G), TP-53 mutation state (Normal-vs.-TP53 mutation, *P* = 1.624E-12) (Figure 4H), Weight (Normal-vs.-weight, *P* = 2.978E-2) (Figure 4I). The expression of CDK2 was related to the survival and prognosis of patients with LUAD (HR = 1.66, *P* = 5.8e-15) (Figure 4J). The higher the expression of CDK2, the shorter the survival time. Through this database, we discovered the CDK2-related genes and the heat maps were shown in Figures 4K,L. Many molecules involved in tumor classical signaling pathways were related to CDK2 expression. PrognoScan database: A new database for meta-analysis of the prognostic value of genes. We further found the expression of CDK2 influenced the OS and RFS of LUAD patients in different GSE datasets (GSE13213: COX *P*-value = 0.027245; GSE31210: COX *P*-value = 0.017966; GSE31210: COX *P*-value = 0.028818; GSE31210: COX *P*-value = 0.004197) (Figures 4M–P).

### The Expression of CDK2 in Pan-Cancer Analysis

**FIGURE 5:**
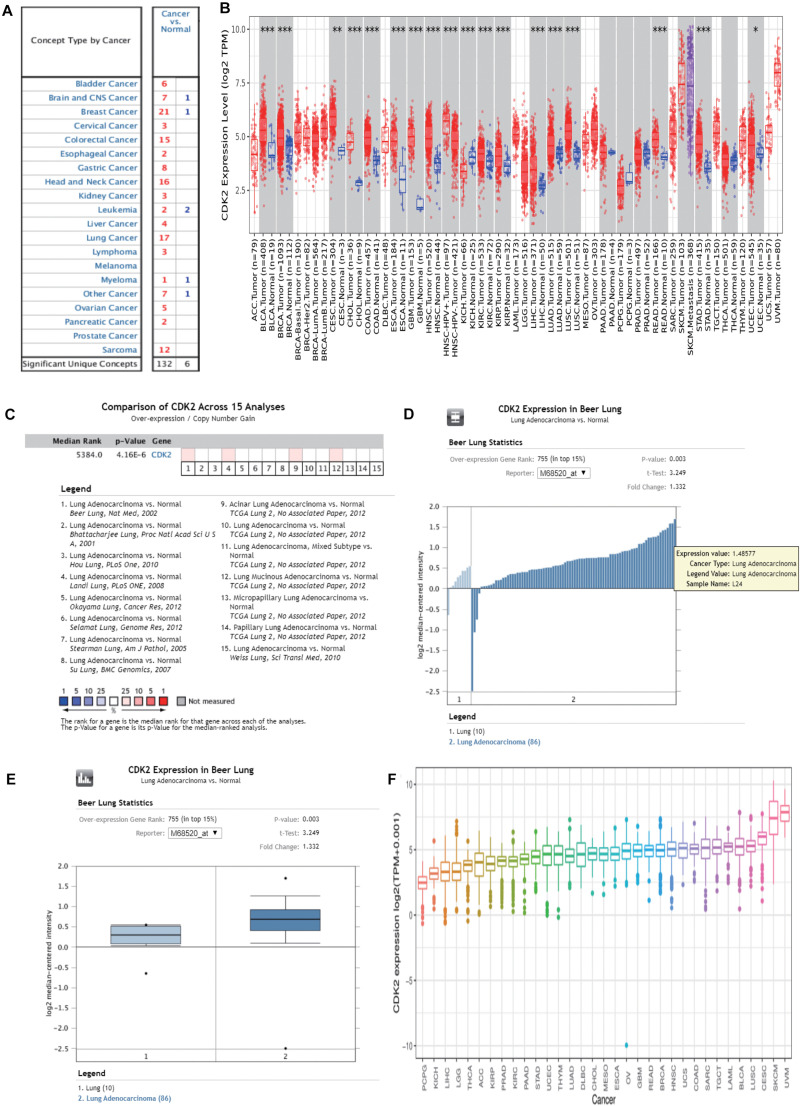
The expression of CDK2 in pan-cancer analysis. **(A)** Through the Oncomine database, the expression of CDK2 was higher in 15 cancer tissues than normal tissues. **(B)** The expression of CDK2 in TIMER database. **(C)** Mata analysis of 15 published studies on LUAD showed that the expression of CDK2 was high in LUAD. **(D,E)** By *t*-test, Box plot and peak plot of CDK2 in LUAD. **(F)** The expression in 33 cancer, a pan-cancer analysis.

**FIGURE 6:**
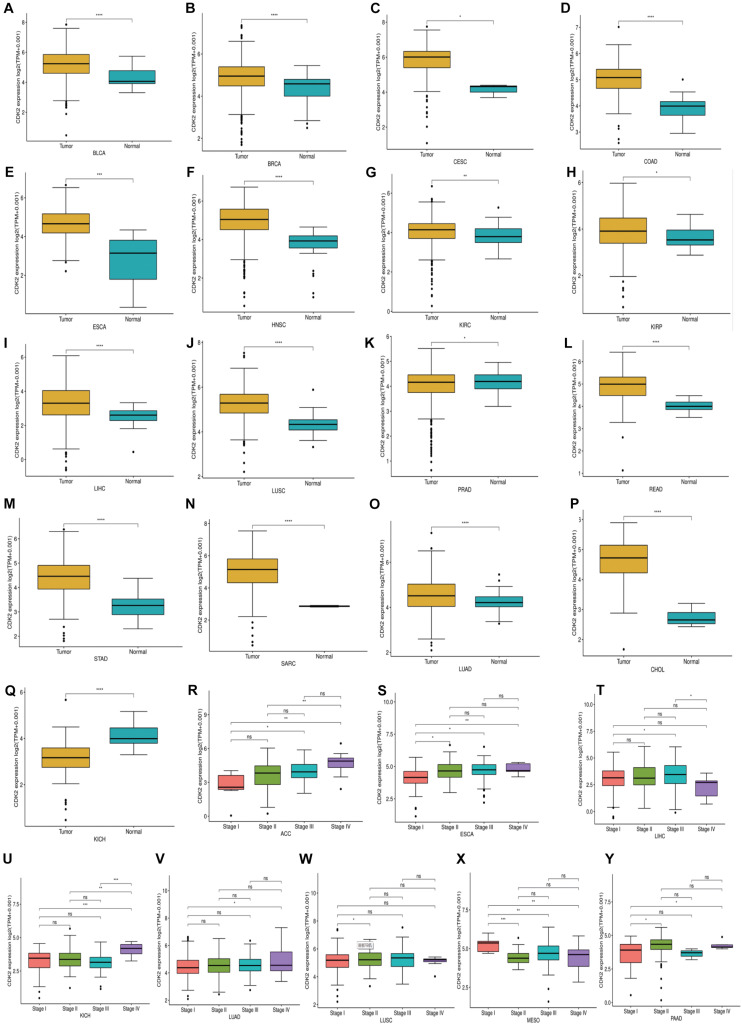
The expression and stage of different cancer. **(A–Q)** The expression of CDK2 was relatively high in 17 cancers. **(R–Y)** The different expression levels of CDK2 had statistical significance on the stage of patients. (*P < 0.05, **P < 0.01, ***P < 0.001, ****P < 0.0001).

**FIGURE 7:**
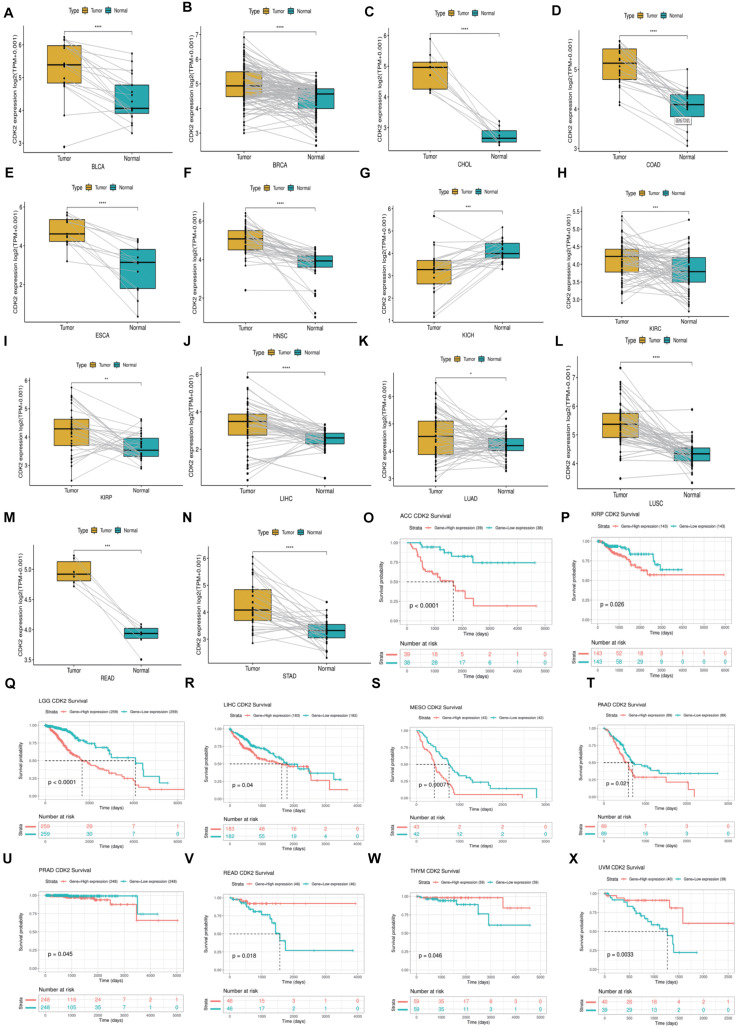
The Paired expression and survival analysis of CDK2. **(A–N)** For different types of cancer, we carried out paired differential expression of CDK2. **(O–X)** In 10 kinds of cancers, the expression of CDK2 was related to the prognosis of patients. (*P < 0.05, **P < 0.01, ***P < 0.001, ****P < 0.0001).

Through the Oncomine database, CDK2 expression was higher in 15 cancer tissues in comparison with normal tissues (Figure 5A). There was a great difference between cancer tissue and normal tissue. The statistical significance between normal and tumor tissues was further found in the TIMER database (the more “^∗^” symbol, the greater the difference) (Figure 5B). Mata analysis of 15 published studies on LUAD showed that expression of CDK2 was higher in LUAD (Median Rank = 5384.0, *P* = 4.16E-6) (Figure 5C). By *t*-test, box plot and peak plot of CDK2 in LUAD were shown in Figures 5D,E (*t*-Test = 3.249, Fold Change = 1.332, *P* = 0.003). Using the R package, we ranked CDK2 by its expression in 33 cancers (Figure 5F). Through different R packages, the expression of CDK2 was relatively higher in 17 cancers (*P* < 0.05) (Figures 6A–Q). The different expression levels of CDK2 had statistical significance on the stage of patients (*P* < 0.05) (Figures 6R–Y). There was significant difference in the expression of CDK2 between stage I and stage III, I and IV, II, and IV LUAD (^∗^/^∗∗^). For different types of cancer, we conducted paired differential expression of CDK2. Simultaneously, we found that there was statistical significance in 14 cancer types (*P* < 0.05) (Figures 7A–N). In 10 cancer types, high CDK2 expression was associated with poor prognosis of the patients (*P* < 0.05) (Figures 7O–X).

### Functional Enrichment Analysis of CDK2

**FIGURE 8:**
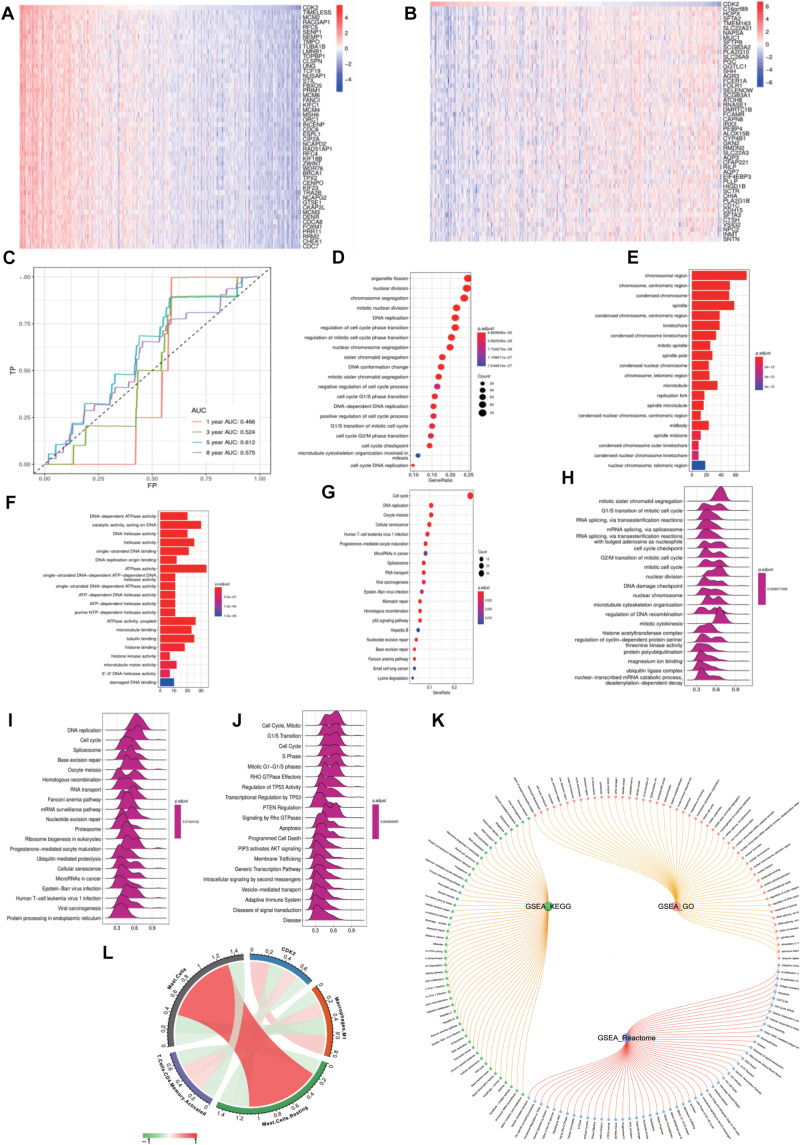
Functional enrichment analysis of CDK2. **(A,B)** The top 50 heat maps of CDK2 positive and negative genes were shown. **(C)** The AUC curve of 1, 3, 5, and 8 years to forecast the survival of patients. **(D–G)** CDK2 was related to the DNA replication, regulation of cell cycle, cell cycle checkpoint, P53 signaling pathway. **(H–J)** Wave charts about GO, KEGG, Reactome showed that CDK2 involved in classic tumor signaling pathways, such as regulation of TP53 activity, PTEN regulation, apoptosis, P13K-AKT signaling pathway. **(K)** The circle graph of pathways. **(L)** CDK2 was positively correlated with CD4 T cells (Cor = 0.1679, *P* = 0.0003), macrophage M1 (Cor = 0.2437, *P* = 1.40E-07) and negatively correlated with Mast cells (Cor = –0.1545, *P* = 0.0009) by CIBERSOPT algorithm.

We compared CDK2 high expression groups with low expression groups in LUAD, and Figures 8A,B showed the top differentially expressed genes in two groups of cancer specimens. We took FP as the abscissa and TP as the ordinate and showed the AUC curve of 1, 3, 5, and 8 years to forecast the survival of patients. The areas under the curve were 0.465, 0.524, 0.612, and 0.575 (Figure 8C). “Clusterprofiler” R package was used to show the function analysis. And CDK2 was related to the DNA replication, regulation of cell cycle, cell cycle checkpoint, P53 signaling pathway. The bubble and bar charts were shown in Figures 8D–G. Wave charts about GO, KEGG, Reactome showed that CDK2 involved in classic tumor signaling pathways, such as regulation of TP53 activity, PTEN regulation, apoptosis, P13K-AKT signaling pathway (Figures 8H–J). The circle graph was shown in Figure 8K. For different immune cells, CDK2 was positively correlated with CD4 T cells (Cor = 0.1679, *P* = 0.0003), macrophage M1 (Cor = 0.2437, *P* = 1.40E-07) and negatively correlated with Mast cells (Cor = –0.1545, *P* = 0.0009) by CIBERSOPT algorithm (Figure 8L).

### Characteristics of CDK2 Immune Cells Infiltration

**FIGURE 9:**
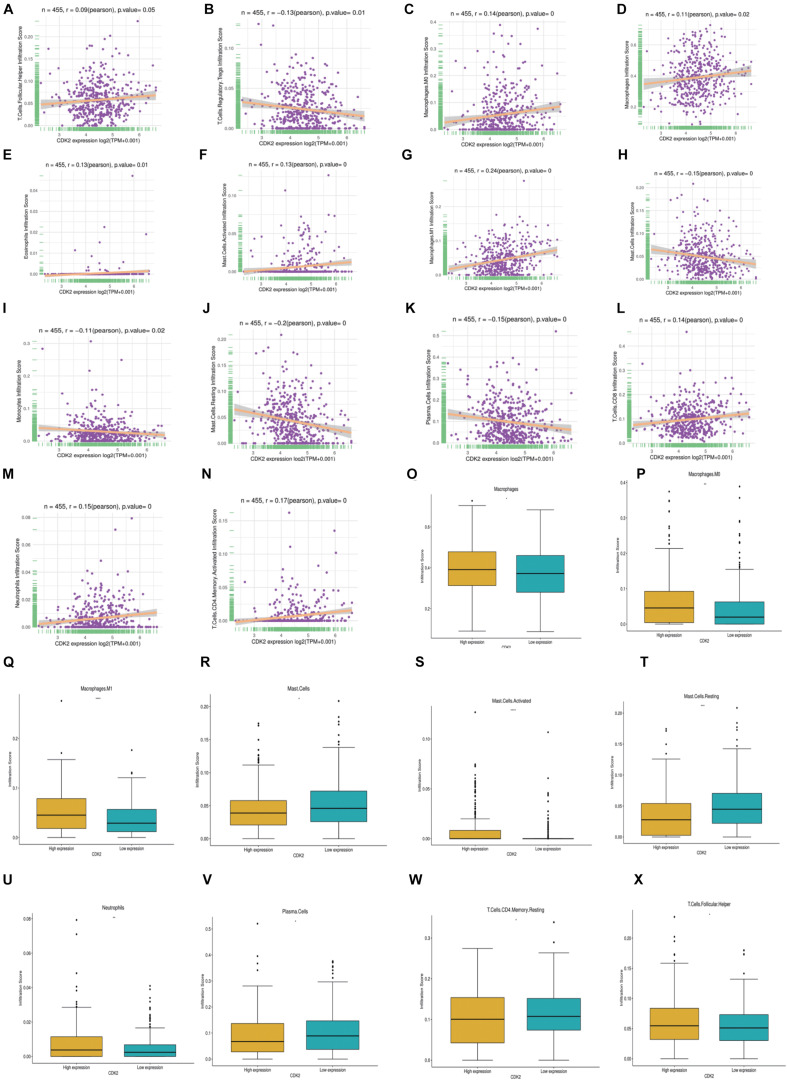
Characteristics of CDK2 immune cells infiltration. **(A–N)** 14 immune cells (T cells Follicular, T cells Regulatory Tregs, Macrophages.M0, Macrophages, Eosinophils, Mast cells activated, Macrophages M1, Mast cells, Monocytes, Mast cells Resting, Plasma cells, T cells CD8, Neutrophils, T cells CD4) were closely related to the expression of CDK2 (*P* ≤ 0.05). **(O–X)** In different CDK2 expression groups, the expression of immune cells had systematic differences (*P* < 0.05).

In LUAD, we further investigated different expression of CDK2 in different immune cell types. We found that 14 immune cells were closely related to CDK2 expression, such as T cells Follicular (*P* = 0.05, r = 0.09), T cells Regulatory Tregs (*P* = 0.01, *r* = –0.13), Macrophages.M0 (*P* = 0, *r* = 0.14), Macrophages (*P* = 0.02, *r* = 0.11), Eosinophils (*P* = 0.01, *r* = 0.13), Mast cells activated (*P* = 0, *r* = 0.13), Macrophages M1 (*P* = 0, *r* = 0.24), Mast cells (*P* = 0, *r* = –0.15), Monocytes (*P* = 0.02, *r* = –0.11), Mast cells Resting (*P* = 0, *r* = –0.2), Plasma cells (*P* = 0, *r* = –0.15), Tcells CD8 (*P* = 0, *r* = 0.14), Neutrophils (*P* = 0, *r* = 0.15), T cells CD4 (*P* = 0, *r* = 0.17) (Figures 9A–N). According to the median value of CDK2 expression, patients with LUAD were divided into the high expression groups and low expression groups. In different groups, the expression of 10 immune cells had systematic differences (*P* < 0.05) (Figures 9O–X).

### Construction of Immune Related Forecasting Model

**FIGURE 10:**
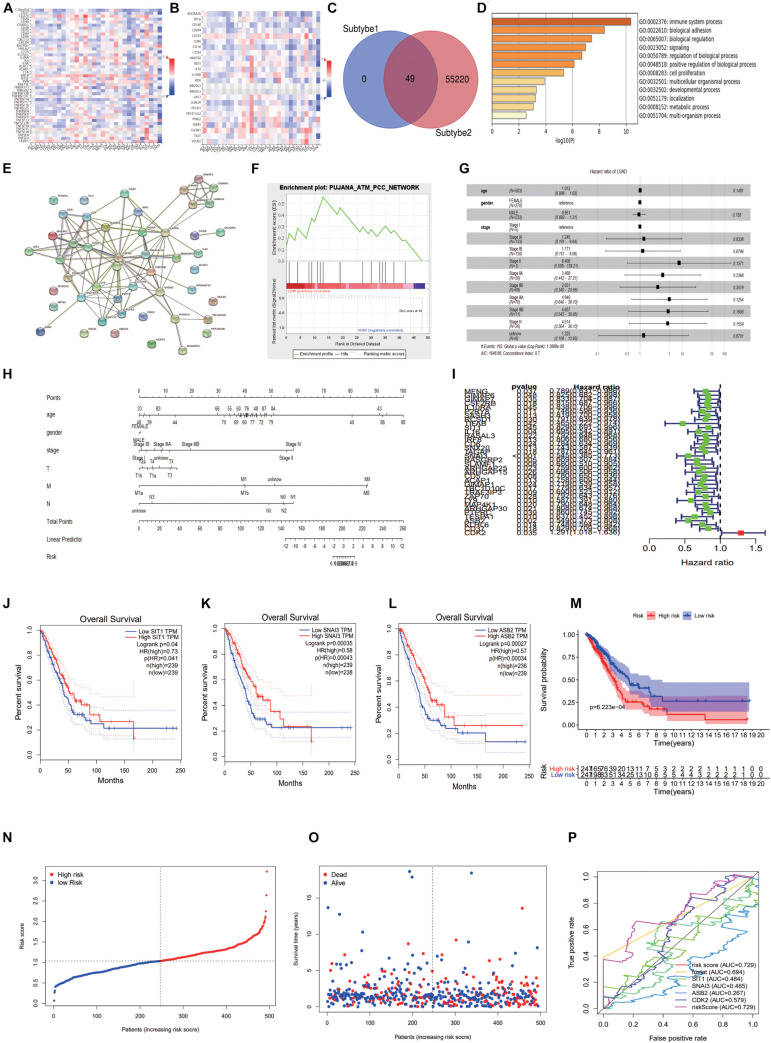
Construction of immune related forecasting model. **(A,B)** The heat maps of immunopotentiators and immunosuppressants about CDK2. **(C)** 49 genes were mixed from the TCGA using “perl” and “R” package. **(D)** The most abundant pathway of 49 genes was immune system process. **(E)** The protein network interaction of these 49 immune related genes. **(F)** GSEA analyzed the function, ES, NES, NOM *p*-val, FDR *q*-val of 49 genes. **(G)** The forest map showed Hazard ratio and concordance index (0.7) of LUAD. **(H)** Nomogram model combined several clinical factors, such as age, gender, stage, T, N, M. **(I)** We showed the Hazard ratio (HR), HR95L, HR95H, p-value of these 36 significant genes. **(J–L)** Through the GEPIA database, SIT1, SNAI3, ASB2 were lower expressed in LUAD. **(M)** The risk curve showed that the high-risk group was more lethal to lung cancer patients. **(N,O)** The high and low risk maps. **(P)** The ROC curve showed the different AUC of four interesting genes.

Through the TISIDB database, we found CDK2-related immunomodulators and the heat maps of immunopotentiators and immunosuppressants were shown in Figures 10A,B. By sorting the *P*-value (*P* < 0.05), we identified 13 immunosuppressants (ADORA2A, BTLA, CD274, CSF1R, IL10, KDR, LAG3, LGALS9, PDCD1, PDCD1LG2, TGFB1, TIGIT, VTCN1) and 21 immunopotentiators (CD27, CD276, CD28, CD40LG, CD48, CD70, CD80, CXCL12, CXCR4, ENTPD1, HHLA2, ICOSLG, IL2RA, IL6, IL6R, KLRC1, MICB, PVR, TMEM173, TNFRSF13B, ULBP1) with a high correlation of CDK2. In the cBioProtal, we explored forty-nine genes associated with 34 immunomodulators. Clinical data and gene expression data were downloaded from TCGA database. Forty-nine genes were mixed from the TCGA using “perl” and “R” package (Figure 10C). Through the Metascape database, the most abundant pathway of 49 genes was the immune system process, other function terms were biological adhesion, biological regulation, cell proliferation (Figure 10D). The BP, CC, MF was showed specifically in Table 2. The protein network interaction (PPI) of these 49 immune related genes were shown in the Figure 10E (*P* < 1.0e-16). GSEA analyzed the function, ES, NES, NOM p-val, FDR q-val of 49 genes. The statistically significant items were PUJANA_ATM_PCC_NETWORK and INTRACELLULAR_SIGNAL_TRANSDUCTION (*P* < 0.01) (Figure 10F and Table 3). Combing with clinical data and expression matrix, univariate and multivariate regression analyses were performed on age, gender and tumor stage (Total *P* = 1.399e-08) (Table 4). The forest map showed HR and concordance index (0.7) of LUAD. As predicted, the tumor stage was an independent risk factor for the prognosis of patients with LUAD (Figure 10G). Nomogram model combined several clinical factors, such as age, gender, stage, T, N, M to intuitively analyze the prognosis of LUAD (Figure 10H). Each patient can be assessed by nomogram model based on baseline clinical data. Univariate regression analysis showed that 36 genes were associated with the prognosis of LUAD (*P* < 0.05). We showed the HR, HR95L, HR95H, *P*-value of these 36 significant genes. CDK2 was an independent prognostic gene in LUAD (HR > 1) (Table 5 and Figure 10I). Multivariate regression analysis was used to analyze the 36 genes and only four genes (SIT1, SNAI3, ASB2, and CDK2) were included in the prediction model (Table 6). Through the GEPIA database, SIT1 (*P* = 0.04), SNAI3 (*P* = 0.00035), ASB2 (*P* = 0.00027) were lower expressed in LUAD (*P* < 0.05) (Figures 10J–L). To verify the prognostic model, the risk curve showed that the high-risk group was more lethal to lung cancer patients (*P* = 6.223E-04) (Figure 10M). Based on different risk scores, patients were divided into high and low risk groups using “R” package (Figures 10N,O). The ROC curve showed the different AUC of 4 interesting genes and CDK2 had more predictive value in the prognostic model (AUC = 0.579) (Figure 10P).

**TABLE 2:** The BP, CC, and MF were showed specifically of 49 immune related genes.

GO term	Subgroup	Enrichment score
Positive regulation of GTPase activity	BP	6.911710228
B cell receptor signaling pathway	BP	28.92678725
Signal transduction	BP	3.699937904
T cell differentiation	BP	39.05116279
Regulation of small GTPase mediated signal transduction	BP	11.65706352
T cell receptor signaling pathway	BP	10.55436832
Positive regulation of T cell proliferation	BP	19.5255814
T cell costimulation	BP	15.019678
Positive regulation of T cell receptor signaling pathway	BP	97.62790698
Innate immune response	BP	4.540832883
G-protein coupled purinergic nucleotide receptor signaling pathway	BP	55.78737542
Adaptive immune response	BP	7.915776241
Positive regulation of Rho protein signal transduction	BP	30.03935599
Response to lipopolysaccharide	BP	7.143505389
Positive regulation of cytosolic calcium ion concentration involved in phospholipase C-activating G-protein coupled signaling pathway	BP	27.89368771
Regulation of defense response to virus by virus	BP	27.89368771
Positive regulation of B cell proliferation	BP	20.02623733
Peptidyl-tyrosine autophosphorylation	BP	19.5255814
Release of sequestered calcium ion into cytosol	BP	19.0493477
Immunological synapse	CC	44.66666667
T cell receptor complex	CC	63.27777778
Membrane	CC	2.070909091
Plasma membrane	CC	1.658335355
Cytosol	CC	1.717948718
Integral component of plasma membrane	CC	2.146525324
Membrane raft	CC	5.529126214
GTPase activator activity	MF	10.75651135
GTP binding	MF	5.861458333
Receptor activity	MF	6.914900154
G-protein coupled purinergic nucleotide receptor activity	MF	53.59047619
Protein tyrosine kinase activity	MF	8.461654135
SH2 domain binding	MF	25.87126437

**TABLE 3:** The GSEA analysis of 49 mRNAs.

Name	Size	ES	NES	NOM *p*-val	FDR *q*-val	FWER *p*-val	Rank at max	Leading edge
PUJANA_ATM_PCC_NETWORK	16	0.5574023	1.7549632	0	0	0	13	Tags = 50%, list = 27%, signal = 46%
LEE_DIFFERENTIATING_T_LYMPHOCYTE	16	0.5064702	1.1931181	0.33333334	0.6333333	0.8	22	Tags = 69%, list = 45%, signal = 84%
SMID_BREAST_CANCER_NORMAL_LIKE_UP	22	0.45970738	0.9803642	0.4	0.4888889	0.8	12	Tags = 36%, list = 24%, signal = 27%
SMID_BREAST_CANCER_LUMINAL_B_DN	15	0.344885	0.68991035	1	0.8833334	1	1	Tags = 13%, list = 2%, signal = 9%
GO_REGULATION_OF_INTRACELLULAR_SIGNAL_ TRANSDUCTION	16	-0.45997676	-1.2494261	0	0	0	2	Tags = 6%, list = 4%, signal = 4%

**TABLE 4:** Univariate and multivariate regression analysis of age, gender, and tumor stage.

	coef	exp(coef)	se(coef)	*z*	*p*
Age	0.011894	1.011965	0.008224	1.446	0.148
genderMALE	–0.050014	0.951216	0.162323	–0.308	0.758
stageStage IA	0.219186	1.245063	1.044445	0.21	0.834
stageStage IB	0.158106	1.17129	1.043788	0.151	0.88
stageStage II	2.128962	8.406135	1.432179	1.487	0.137
stageStage IIA	1.243594	3.468057	1.051101	1.183	0.237
stageStage IIB	0.9751	2.651431	1.047535	0.931	0.352
stageStage IIIA	1.597291	4.939634	1.042318	1.532	0.125
stageStage IIIB	1.538441	4.657324	1.096495	1.403	0.161
stageStage IV	1.50713	4.51376	1.060871	1.421	0.155
*P* = 1.399e-08					

**TABLE 5:** The analysis of Univariate regression about 36 genes.

Id	HR	HR.95L	HR.95H	*P*-value
MFNG	0.788791335	0.631264471	0.985627735	0.036858598
GIMAP6	0.825241244	0.68227954	0.998158484	0.04782116
GIMAP7	0.833265382	0.7035123	0.98694962	0.034680373
CSF2RB	0.814678719	0.687175564	0.965839662	0.018264635
IL10RA	0.838397617	0.705873794	0.995802041	0.044655471
P2RY8	0.746446286	0.595538414	0.935593818	0.011158897
SASH3	0.819264713	0.700490753	0.958177773	0.01261093
RCSD1	0.790672034	0.639423589	0.977696595	0.030144597
TIFAB	0.465083695	0.222080992	0.973981795	0.042370157
SIT1	0.829408759	0.690741005	0.995914365	0.045088923
IL16	0.694821369	0.541782159	0.89109013	0.004125937
RASAL3	0.791834804	0.648056113	0.967512448	0.022429427
IRF8	0.805982708	0.679749247	0.955658471	0.013071014
CD6	0.783523435	0.633680321	0.968799176	0.024280529
SNX20	0.742751039	0.587258851	0.939413865	0.013084672
TAGAP	0.786867105	0.644621205	0.960501822	0.018468635
SNAI3	0.545360728	0.384584195	0.773350355	0.000668422
RASGRP2	0.669309183	0.506911612	0.883733519	0.004630788
SLAMF1	0.679757859	0.510377564	0.905350821	0.008290951
ARHGAP25	0.759490747	0.599527918	0.962134	0.022615883
ARHGAP15	0.696431097	0.506489419	0.957603961	0.025975442
CD5	0.779912525	0.649789891	0.936092658	0.007605839
ACAP1	0.758479625	0.609407945	0.944016806	0.013287558
GIMAP1	0.718737917	0.539208263	0.958042056	0.024304367
TBC1D10C	0.778901559	0.634277612	0.956501738	0.01711004
TRAF3IP3	0.69040004	0.522777097	0.911769505	0.009030209
ZAP70	0.792228171	0.643311406	0.975616893	0.028356063
LY9	0.586557529	0.391159073	0.879564757	0.009858385
MAP4K1	0.790353869	0.64826384	0.963587971	0.019976631
ARHGAP30	0.808036247	0.674219373	0.968412661	0.021030946
PTPRC	0.859646397	0.744782085	0.992225703	0.038770457
TESPA1	0.637294984	0.452324142	0.897906742	0.010006127
ASB2	0.549252388	0.373292675	0.808154581	0.002358241
KLHL6	0.747994992	0.593856228	0.942141348	0.013656291
CD37	0.828826076	0.709387822	0.968373918	0.018042429
CDK2	1.290598521	1.017922999	1.636316836	0.035146972

**TABLE 6:** The analysis of multivariate regression about four genes.

id	coef	HR	HR.95L	HR.95H	*P*-value
SIT1	0.255264409	1.290802876	0.925388716	1.800510461	0.132760088
SNAI3	–0.400450665	0.670018024	0.422135602	1.063459586	0.089332143
ASB2	–0.799324198	0.449632724	0.219800019	0.919788756	0.028601817
CDK2	0.203710988	1.22594379	0.967951804	1.552699391	0.09107145

### HPA Analysis and Construction of Predictive ceRNA Network in LUAD

**FIGURE 11:**
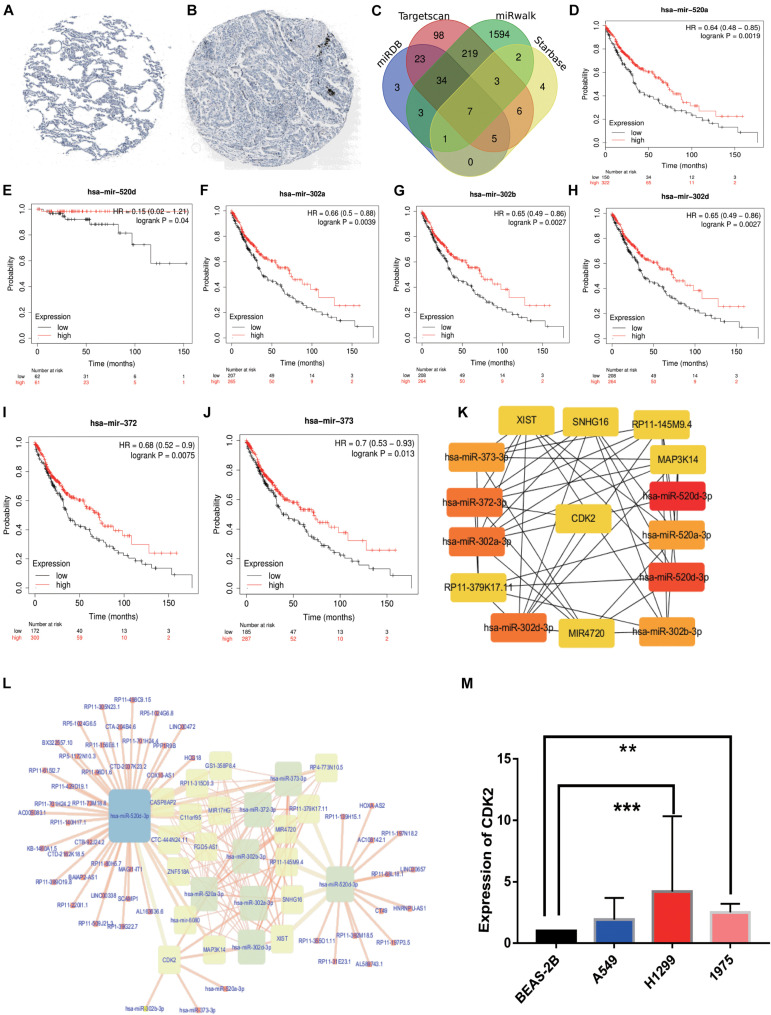
HPA analysis and ceRNA network. **(A)** Immunohistochemical results in normal lung tissues. **(B)** Immunohistochemical results in LUAD tissues. **(C)** The intersection results of the four databases were shown by Venn diagram. **(D–J)** The survival analysis of seven miRNAs. **(K)** 6lncRNAs-7miRNAs-CDK2 were considered as top 15 genes by CytoHubba**. (L)** ceRNA network was constructed by Cytoscape. **(M)** The PCR results about CDK2 expression in BEAS-2B, A549 (*P* = 0.0808), H1299 (*P* = 0.0006), H1975 (*P* = 0.0030). (**P < 0.01, ***P < 0.001).

Immunohistochemical result showed that the CDK2 expression in LUAD tissues was significantly higher than that in normal lung tissues (Figures 11A,B). We selected 455 miRNAs related to CDK2 from TargetScan database. 10,018 miRNAs were found in miRWalk database. And we explored 76 miRNAs from mirDB and 28 miRNAs from Starbase. Combining with differentially expressed miRNAs in LUAD, 7 miRNAs were joined in the network using Venn map, such as hsa-miR-302b-3p, hsa-miR-372-3p, hsa-miR-302a-3p, hsa-miR-373-3p, hsa-miR-520a-3p, hsa-miR-520d-3p, and hsa-miR-302d-3p (Figure 11C). These miRNAs had obvious influence on the prognosis of LUAD patients (*P* < 0.05) (Figures 11D–J). We used the Starbase database to find the potential lncRNAs that regulate seven miRNAs. These coding genes and non-coding genes were interacted with each other by using Cytoscape software (Figure 11L). According to the degree in the network, we screened out the top 15 genes using CytoHubba (six lncRNAs: XIST, SNHG16, RP11-145M9.4, MAP3K14, MIR4720, and RP11-379K17.11) (Figure 11K).

### The PCR Results of CDK2 Expression

Compared with the expression of CDK2 in BEAS-2B cell line, the expression of CDK2 in A549 cell line was increased, but there was no statistical difference (*P* = 0.0808). There were significant differences between H1299 cell line (*P* = 0.0006) and H1975 cell line (*P* = 0.0030) (Figure 11M).

### Correlation Analysis of Drug Resistance

**FIGURE 12:**
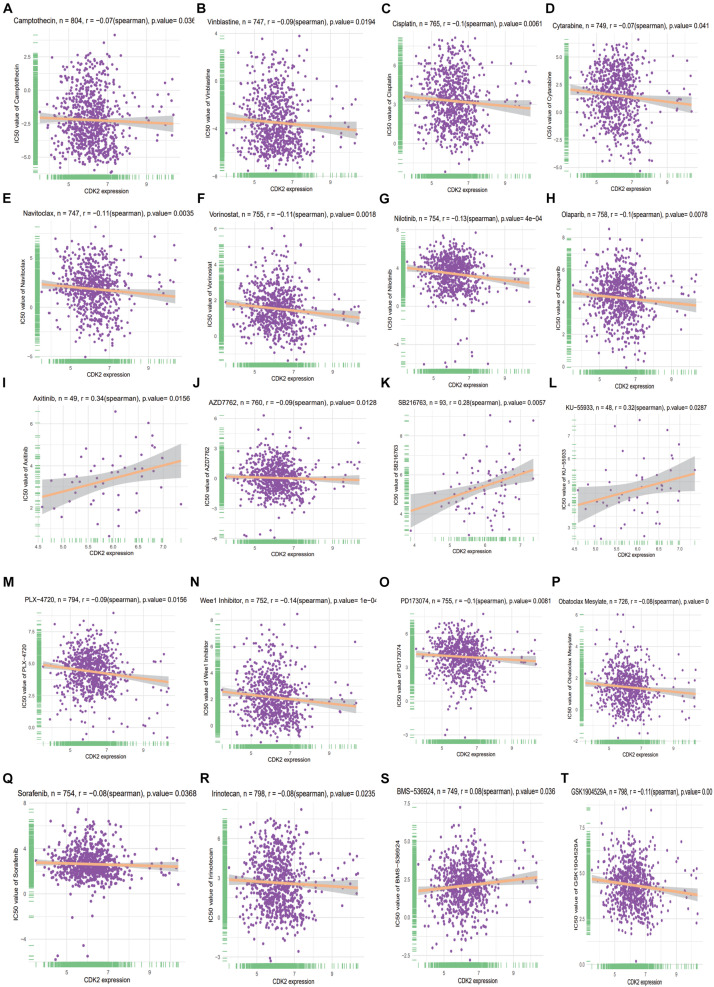
The top 20 anti-tumor drug resistance studies related to CDK2 **(A–T)**.

A total of 192 anti-tumor drugs were included in the study. The IC50 level of 89 anti-tumor drugs were related to the expression of CDK2. According to the size of *P* value (*P* < 0.05), we screened out the top 20 anti-tumor drugs with positive or negative correlation, such as Camptothecin (*r* = –0.074, *P* = 0.000018), Vinblastine (*r* = –0.085, *P* = 0.0000243), Cisplatin (*r* = –0.099, *P* = 0.0000843), Cytarabine (*r* = –0.0746, *P* = 0.0000975), Navitoclax (*r* = –0.106, *P* = 0.000158), Vorinostat (*r* = –0.113, *P* = 0.0002), Nilotinib (*r* = –0.127, *P* = 0.000258), Olaparib (*r* = –0.0966, *P* = 0.000302), Axitinib (*r* = 0.343, *P* = 0.000381), AZD7762 (*r* = –0.0902, *P* = 0.000382), SB216763 (*r* = 0.284, *P* = 0.000403), KU-55933 (*r* = 0.315, *P* = 0.000404), PLX-4720 (*r* = –0.0857, *P* = 0.000428), Wee1 Inhibitor (*r* = –0.141, *P* = 0.000780), PD173074 (*r* = –0.0963, *P* = 0.000794), Obatoclax Mesylate (*r* = –0.08009, *P* = 0.000872), Sorafenib (*r* = –0.076, *P* = 0.0009), Irinotecan (*r* = –0.0801, *P* = 0.00120), BMS-536924 (*r* = 0.0765, *P* = 0.0012), and GSK1904529A (*r* = –0.113, *P* = 0.0012) (Table 7 and Figures 12A–T).

**TABLE 7:** Here are the top 20 anti-tumor drug resistance studies related to CDK2.

Correlation	*p* value	Type	Label
–0.073624414	1.88E-05	Camptothecin	Negative
–0.085496637	2.44E-05	Vinblastine	Negative
–0.099132332	8.43E-05	Cisplatin	Negative
–0.074684535	9.76E-05	Cytarabine	Negative
–0.106844461	0.000158572	Navitoclax	Negative
–0.11367658	0.000200639	Vorinostat	Negative
–0.127941152	0.000258313	Nilotinib	Negative
–0.096615555	0.000302571	Olaparib	Negative
0.343877551	0.000381858	Axitinib	Positive
–0.090294733	0.000382049	AZD7762	Negative
0.284802005	0.000403878	SB216763	Positive
0.315892314	0.00040404	KU-55933	Positive
–0.085796235	0.00042863	PLX-4720	Negative
–0.141613081	0.000780249	Wee1 Inhibitor	Negative
–0.09631745	0.000794901	PD173074	Negative
–0.080096541	0.000872875	Obatoclax Mesylate	Negative
–0.076063235	0.000903871	Sorafenib	Negative
–0.080170909	0.001206821	Irinotecan	Negative
0.076522222	0.00126849	BMS-536924	Positive
–0.113717172	0.001291814	GSK1904529A	Negative

## Discussion

In recent years, the role of immune invasion in the progression of LUAD has been improved. Immunotherapy can resist tumor cells by activating the activity of immune molecules. It is necessary to find effective immune related-markers to predict prognosis and apply the individual therapy. And the study of TME is the key to overcome drug resistance (Li et al., 2020).

Our study first explored the prognostic molecule CDK2, which was differently expressed in LUAD tissues and adjacent non-LUAD tissues (*P* = 1.624E-12). High expression of CDK2 in LUAD has poor prognosis. In order to verify CDK2 in all kinds of cancers, we carried out a pan-cancer study. We further showed the expression, stage analysis, paired expression, survival condition in 33-cancer. The expression, stage and prognosis of CDK2 were obviously different in many cancers. According to published articles, a gene marker is no longer limited to one cancer, but is extended to various cancers, so the credibility has been improved than before. Based on CDK2, we searched for relevant immunomodulators and combined with the clinical expression data of LUAD to conduct univariate and multivariate regression models. Then the Nomogram forecast model of age, gender, stage, T, N, M provided the clinical significance and prognosis. The patient’s condition will be assessed according to the total risk score. Nomogram has a great effect on the prognosis of patients with LUAD. Finally, multivariate regression analysis showed that four mRNAs (SIT1, SNAI3, ASB2, and CDK2) were important for this immune model and significant for the prognosis of patients (*P* < 0.05). CDK2 was a risk molecule with independent prognosis (HR = 1.291, *P* = 0.035). This provides potential markers for targeted therapy with LUAD.

Cell-dependent kinases (CDKs) are involved in proliferation, DNA damage repair (Lim and Kaldis, 2013) and treatment of various tumors (Lin et al., 2021; Majumdar et al., 2021). A variety of mechanisms, including chaperone, positive phosphorylation and negative phosphorylation (Lee et al., 2021) regulate the activity of CDK family. Recently, CDK family molecules have been used as novel molecular markers for tumor-targeted therapy. As a star molecule, CDK2 (Kawakami et al., 2020) participates in classic pathways in various cancers, such as colorectal cancer (Somarelli et al., 2020), neuroblastoma (Poon et al., 2020), breast cancer (Hur et al., 2020), hepatocellular carcinoma (Hou et al., 2019), and prostate cancer (Washino et al., 2019). However, there are few studies on the mechanism of CDK2 in lung cancer. We studied the relationship between CDK2 and tumor immunity, providing a new idea for immunotherapy about LUAD.

In this study, we found that the expression of CDK2 was related to various immune cells including T cells (*P* = 0.05), Macrophages (*P* = 0), Eosinophils (*P* = 0.01), Mast cells (*P* = 0). Therefore, CDK2 can affect the tumorigenesis and proliferation by regulating the mechanism of immune inflammation. Combined with results of COX regression, target molecule CDK2 provides prognosis analysis and treatment strategy.

Cell cycle imbalance is common in different tumors (Chen T. et al., 2021). Cell-dependent kinase (CDK) is involved in many tumors related biological processes, such as cell cycle, immune checkpoint (Hume et al., 2020), RNA transcription (Al-Sanea et al., 2021), cell proliferation (Liu J. et al., 2021). Recently, there have been studies on CDK4 (Pandey et al., 2021) and CDK6 (Pandey et al., 2021), but the mechanism of CDK2 (El-Sattar et al., 2021) in cancer is relatively lacking. In our study, we deeply studied CDK2 and constructed the related immune prediction model. And CDK2 was associated in the P13K-AKT (Li Z. et al., 2021) signaling pathway in LUAD, inducing cell proliferation (Wu et al., 2020). Combing with clinical data, we constructed the forest map and Nomogram model (Cao et al., 2021b; Jang et al., 2021; Wang R. R. et al., 2021; Wang K. et al., 2021) of clinical characteristics. Through the features of patients, we can calculate the total score to evaluate the prognosis of patients. The prognosis of patients can be evaluated by a simple calculation method.

Compared with the current studies, the advantage is that we first established CDK2 related immune forecast model through the univariate, multivariate regression analysis and different “R” packages. This forecast was significant for LUAD patients. And the molecule CDK2 was related to various immune cells and may regulate mechanism in some way. The correlation between CDK2 and immune cells was listed by box diagram and point diagram. Compared with previous studies, they may research some markers in only signal cancer. But we explored detailed information of CDK2 in 33-cancer, a pan-cancer analysis was shown in our study. In various cancers, CDK2 has corresponding systematic significance with several clinical aspects (*P* < 0.05). CDK2 was associated with clinical stage, age, pathological type, TP-53 mutation, smoking with LUAD. According to the risk score, LUAD patients were divided into the high-risk group and low-risk group. There was a great difference between different risk curves (Yu et al., 2021). The survival time of the higher risk group was shorter than that of the lower risk group (*P* = 6.223E-04). This indicates that the immune related model is of great significance for the prognosis of patients with LUAD. All data were collected from reliable GEO database, TCGA, UCSC Xena and this increases the reliability of the data. In recent years, ceRNA network has certain significance in various cancers. The functions of many non-coding genes have been gradually explored (Yuan et al., 2014). Seven miRNAs (hsa-miR-302b-3p, hsa-miR-372-3p, hsa-miR-302a-3p, hsa-miR-373-3p, hsa-miR-520a-3p, hsa-miR-520d-3p, and hsa-miR-302d-3p), six lncRNAs (XIST, SNHG16, RP11-145M9.4, MAP3K14, MIR4720, and RP11-379K17.11) were considered as potential biomarkers in LUAD. Combined with the current literature, these non-coding genes have been studied in other cancers, but less in LUAD. This study provides potential therapeutic targets for LUAD and contributes to immunotherapy.

In recent years, nomogram model has been considered as a tool to predict tumor prognosis (Balachandran et al., 2015; Huang et al., 2016), such as colorectal cancer (Huang et al., 2016) and cervical cancer (Rose et al., 2015). Nomogram model meets our desire for biologically and clinically integrated models. It is not limited to one factor, but combines with various influencing factors to evaluate the prognosis of patients. We constructed nomogram model of age, gender, stage, T, N, M. According to the corresponding score of each influencing factor, the risk index of LUAD patients is estimated to evaluate the survival time.

According to our anti-tumor drug resistance research findings, the IC50 of four drugs (Axitinib, SB216763, KU-55933, BMS-536924) were positive with expression of CDK2. This indicates that cancer patients with high expression of CDK2 are prone to resistance to the above four drugs. Other 16 drugs were negative with the expression of CDK2. The patients with high expression of CDK2 had good response to the above 16 drugs and low resistance rate. Three drugs Cisplatin, Cytarabine, Nilotinib are considered as classic anti-cancer drugs. They are effective for patients with high expression of CDK2 cancer and it is not easy to cause drug resistance. Of course, the mechanism between CDK2 and drug resistance still needs to further be studied.

There are disadvantages in our study compared with the current articles. The target molecules lack of experimental verification and big data support. The regulatory mechanism of target genes is unclear.

In summary, immune-related forecast model was constructed by univariate regression and multivariate regression analysis to guide prognosis of LUAD. K–M curve verified the high-risk group had a poor prognosis (*P* < 0.01). Because of the correlation of CDK2 and various immune cells, CDK2 may be involved in tumor regulation of immune infiltration. CDK2 provides a new immunotherapy target for LUAD, which can improve the prognosis. The construction of ceRNA network provides a new way for exploring potential gene markers with LUAD. Drug resistance research can help patients choose a reasonable treatment plan, not blindly targeted therapy or immunosuppressive therapy.

## Conclusion

In conclusion, we discovered a set of four genes, including expression of CDK2, has a significant prognostic value in LUAD. CDK2 expression is highly associated with immune responses in the cancer. We made some prediction to link CDK2 expression with drug responses and miRNA expression.

## Data Availability Statement

The datasets presented in this study can be found in online repositories. The names of the repository/repositories and accession number(s) can be found in the article/supplementary material.

## Author Contributions

T-TL and RL conceived and designed the study. CH, J-PL, JY, and X-LJ collected the literature. T-TL drafted the manuscript. Y-QQ revised the manuscript. All the authors read and approved the final manuscript.

## Conflict of Interest

The authors declare that the research was conducted in the absence of any commercial or financial relationships that could be construed as a potential conflict of interest.

## Publisher’s Note

All claims expressed in this article are solely those of the authors and do not necessarily represent those of their affiliated organizations, or those of the publisher, the editors and the reviewers. Any product that may be evaluated in this article, or claim that may be made by its manufacturer, is not guaranteed or endorsed by the publisher.

## Footnotes

1
https://www.ncbi.nlm.nih.gov/gds/?term

2
https://xena.ucsc.edu/

3
https://www.cancerrxgene.org/

4
https://metascape.org/gp/index.html#/main/step1

5
https://www.kegg.jp/

6
https://david.ncifcrf.gov/tools.jsp

7
http://ualcan.path.uab.edu/index.html

8
http://dna00.bio.kyutech.ac.jp/PrognoScan/index.html

9
https://www.oncomine.org/resource/login.html

10
https://cistrome.shinyapps.io/timer/

11
https://www.gsea-msigdb.org/gsea/msigdb

12
https://cibersort.stanford.edu/index.php

13
http://cis.hku.hk/TISIDB/

14
https://www.cbioportal.org/

15
https://string-db.org/cgi/network

16
http:/www.targetscan.org

17
http://mirwalk.umm.uni-heidelberg.de/

18
http://mirdb.org/

19
http://starbase.sysu.edu.cn/starbase2/index.php

20
http://bioinformatics.psb.ugent.be/webtools/Venn/

21
https://www.graphpad.com/

22
http://gepia.cancer-pku.cn/index.html

## Abbreviations

TME: tumor microenvironment
ceRNA: competing endogenous RNA
LUAD: lung adenocarcinoma
GEO: Gene Expression Omnibus
TCGA: The Cancer Genome Atlas
GDSC: Genomics of Drug Sensitivity in Cancer
DEG: differentially expressed genes
GSEA: Gene Set Enrichment Analysis
LUSC: lung squamous cell carcinoma
NSCLC: non-small cell lung cancer
SII: systemic immune-inflammation index
CDK: cell-dependent kinases
BP: biological process
CC: cellular component
MF: molecular function
OS: The Overall Survival
RFS: Relapse-Free Survival
FP: false positive
TP: true positive
KEGG: Kyoto Encyclopedia of Genes and Genomes
AML: acute myeloid leukemia
GO: Gene Ontology
HPA: Human Protein Atlas
PCR: polymerase chain reaction.
